# Cyanidin Stimulates Insulin Secretion and Pancreatic β-Cell Gene Expression through Activation of l-type Voltage-Dependent Ca^2+^ Channels

**DOI:** 10.3390/nu9080814

**Published:** 2017-07-28

**Authors:** Tanyawan Suantawee, Sara T. Elazab, Walter H. Hsu, Shaomian Yao, Henrique Cheng, Sirichai Adisakwattana

**Affiliations:** 1 Program in Biomedical Sciences, Graduate School, Chulalongkorn University, Bangkok 10330, Thailand; suantawee@gmail.com; 2 Department of Pharmacology, Faculty of Veterinary Medicine, Mansoura University, Mansoura 35516, Egypt; sara.taha@ymail.com; 3 Department of Biomedical Sciences, College of Veterinary Medicine, Iowa State University, Ames, IA 50011, USA; whsu@iastate.edu; 4 Department of Comparative Biomedical Sciences, School of Veterinary Medicine, Louisiana State University, Baton Rouge, LA 70803, USA; shaomia@lsu.edu (S.Y.); hcheng@lsu.edu (H.C.); 5 Department of Nutrition and Dietetics, Faculty of Allied Health Sciences, Chulalongkorn University, Bangkok 10330, Thailand

**Keywords:** cyanidin, insulin secretion, pancreatic β-cells, voltage-dependent Ca^2+^ channel, gene expression

## Abstract

Cyanidin is a natural anthocyanidin present in fruits and vegetables with anti-diabetic properties including stimulation of insulin secretion. However, its mechanism of action remains unknown. In this study, we elucidated the mechanisms of cyanidin for stimulatory insulin secretion from pancreatic β-cells. Rat pancreatic β-cells INS-1 were used to investigate the effects of cyanidin on insulin secretion, intracellular Ca^2+^ signaling, and gene expression. We detected the presence of cyanidin in the intracellular space of β-cells. Cyanidin stimulated insulin secretion and increased intracellular Ca^2+^ signals in a concentration-dependent manner. The Ca^2+^ signals were abolished by nimodipine, an l-type voltage-dependent Ca^2+^ channel (VDCC) blocker or under extracellular Ca^2+^ free conditions. Stimulation of cells with cyanidin activated currents typical for VDCCs and up-regulated the expression of glucose transporter 2 (GLUT2), Kir_6.2_, and Cav_1.2_ genes. Our findings indicate that cyanidin diffuses across the plasma membrane, leading to activation of l-type VDCCs. The increase in intracellular Ca^2+^ stimulated insulin secretion and the expression of genes involved in this process. These findings suggest that cyanidin could be used as a promising agent to stimulate insulin secretion.

## 1. Introduction

Glucose is the main physiological stimulus for insulin secretion from pancreatic β-cells. When glucose enters cells through glucose transporter 2 (GLUT2), it is phosphorylated to glucose-6 phosphate by glucokinase and metabolized to generate ATP. The generation of ATP induces the closure of ATP-sensitive K^+^ channels (K_ATP_), which evokes membrane depolarization and the opening of voltage-dependent Ca^2+^ channels (VDCCs). This increases intracellular Ca^2+^ concentration and stimulates insulin secretion [1,2]. In type 2 diabetes mellitus (T2DM), the failure of pancreatic β-cells results from chronic exposure to a high level of circulating glucose, which causes insufficient insulin secretion or loss of insulin action at target tissues [3]. Current treatments for hyperglycemia include oral hypoglycemic agents such as sulfonylureas and insulin secretagogues that stimulate insulin release from β-cells. However, these compounds can result in significant hypoglycemia and secondary insulin secretion failure [4]. To overcome this issue, several studies suggest the use of phytochemical compounds from fruits, vegetables, and herbal medicines to induce insulin secretion [5,6,7].

Cyanidin, a member of the anthocyanidin class, is one of the most abundant flavonoids found in red-purple diet sources such as fruits, vegetables, and red wine [8]. Cyanidin and its derivatives possess several pharmacological properties including antioxidant [9], anti-inflammatory [10], and antihyperglycemic activity [11,12]. For example, we demonstrated the ability of cyanidin and its derivatives to inhibit intestinal α-glucosidase and pancreatic α-amylase, which are key enzymes for carbohydrate digestion and absorption [11]. Furthermore, supplementation of cyanidin-derivative enriched extract prevents the progression of diabetes by preserving pancreatic islet architecture in streptozotocin (STZ)-induced diabetic rats [12]. Cyanidin and its derivatives also suppress hyperglycemia and improve glucose homeostasis and insulin sensitivity [13,14]. Our previous study indicates that cyanidin is an anti-glycating agent against glucose- and methylglyoxal (MG)-induced protein glycation/oxidation by MG trapping and free radical scavenging. Therefore, cyanidin could be used as a phytochemical compound to delay and prevent diabetic complications [15]. Cyanidin induces insulin secretion under low and high glucose conditions [16]. However, the mechanism by which cyanidin stimulates insulin secretion from pancreatic β-cells is unknown.

The purpose of this study was to elucidate the signaling pathway by which cyanidin induces insulin secretion from pancreatic β-cells. In the present study, cyanidin itself has ability to stimulate insulin secretion from pancreatic β-cells by increasing intracellular calcium through l-type Ca^2+^ channels. Following supplementation, cyanidin exhibits gene-regulatory activity by up-regulating expression of gene-associated insulin secretion. These findings indicate that cyanidin can be considered as a promising agent for stimulating insulin secretion and regulating gene transcriptions related to insulin secretory response.

## 2. Materials and Methods 

### 2.1. Chemicals

All reagents were purchased from Sigma Chemical Co. (St. Louis, MO, USA), except Fura-2 acetoxymethyl ester (Fura-2AM) and Naturstoff reagent A, which were purchased from Cayman Chemical Co. (Ann Arbor, MI, USA) and Alfa Aesar (Ward Hill, MA, USA), respectively. Naturstoff reagent A was dissolved in ethanol at the concentration of 5% and then in phosphate buffered saline (PBS) at the concentration of 0.2%. Cyanidin chloride was synthesized from quercetin according previous method [17]. Cyanidin was dissolved in DMSO to obtain the desired concentrations.

### 2.2. Cell Culture

Pancreatic β-cells INS-1 were used in the study and cultured in RPMI-1640 medium containing 11 mM glucose supplemented with 10% fetal bovine serum (FBS), 2 mM l-glutamine, 1 mM sodium pyruvate, and 50 µM 2-mercaptoethanol. The cells were maintained in humidified atmosphere at 5% CO_2_ at 37 °C. All experiments were performed with cells between passages 70 and 85.

### 2.3. Cyanidin Localization

The visualization of cyanidin was determined using Naturstoff reagent A with minor modification [18]. Briefly, INS-1 cells were grown on 25 mm coverslips and treated with 100 µM cyanidin or DMSO (negative control). After 10 min of incubation, cells were washed with PBS and incubated with 0.2% Naturstoff reagent A for 5 min. Subsequently, cells were washed again with PBS and fixed in 4% paraformaldehyde for 10 min. Finally, the coverslips were placed on glass slides and examined by confocal laser scanning microscope. Fluorescent images were analyzed at an excitation wavelength of 488 nm and emission wavelengths of 620–705 nm.

### 2.4. Static Incubation for Insulin Determination

INS-1 cells were cultured on 24-well plates at a density of 5 × 10^5^ cells/well. Upon reaching confluency, the cells were incubated for 30 min in modified Krebs-Ringer bicarbonate buffer (KRB) containing 136 mM NaCl, 4.8 mM KCl, 2.5 mM CaCl_2_, 1.2 mM KH_2_PO_4_, 1.2 mM MgSO_4_, 5 mM NaHCO_3_, 10 mM HEPES, 4 mM glucose, and 0.1% bovine serum albumin (BSA), pH 7.4. The cells were then incubated for 30 min in KRB containing different cyanidin concentrations or KCl (positive control). After incubation, the supernatant was collected and stored frozen at −80 °C for insulin determination with radioimmunoassay (RIA). Finally, the number of cells/well was quantified to normalize the insulin concentrations. Each treatment was done in quadruplicates and repeated three times.

### 2.5. Real-Time Ca^2+^ Imaging Analysis

Cells were grown on round glass coverslip for 48 h until 80–90% confluent and loaded with 5 µM Fura-2AM for 30 min at 37 °C. A Ca^2+^ imaging buffer containing in mM: 136 NaCl, 4.8 KCl, 1.2 CaCl_2_, 1.2 MgSO_4_, 10 HEPES, 4 glucose, and 0.1% BSA at a pH of 7.4 was used for Fura-2AM loading and perfusion throughout the experiments, except in Ca^2+^ free experiments where CaCl_2_ was absent and 0.1 mM Ethylene glycol-bis(2-aminoethylether)-*N,N,N′,N′*-tetraacetic acid (EGTA) added. Intracellular Ca^2+^ signals were recorded using a dual excitation fluorometric imaging system (TILL-Photonics Gräfelfingen, Germany) controlled by TILLvisION software. Fura-2AM loaded cells were excited at wavelengths of 340 nm and 380 nm. Fluorescence emissions were sampled at 1 Hz and computed as F340/F380 ratio. The data were plotted as the averages from several cells from three independent experiments or the peak Ca^2+^ increases.

### 2.6. Electrophysiology

INS-1 cells were grown on round glass coverslips for 18 h and transferred to the patch clamp recording chamber. Cells were maintained in external solution containing in mM: 102 NaCl, 10 CaCl_2_, 5.4 CsCl, 1 MgCl_2_, 20 TEA-Cl, 5 HEPES, and 10 glucose (pH 7.4 with NaOH). The pipette solution contained in mM: 135 CsCl, 3 MgCl_2_, 3 Mg-ATP, 10 EGTA, and 5 HEPES (pH 7.4 with CsOH). VDCC currents were recorded in the tight-seal whole-cell configuration mode at 21–25 °C. High-resolution current recordings were acquired by a computer-based patch-clamp amplifier system (EPC-10, HEKA, Lambrecht, Germany). Patch pipettes had resistances of 3–5 MΩ. After break-in, 50 ms voltage ramps spanning from −100 mV to +100 mV were applied every 2 s from a holding potential (Vh) of −80 mV until the currents reached a steady amplitude. Then cells were clamped from Vh = −80 mV to membrane potentials of −80 mV to +80 mV in 10 mV steps of 400 ms each with 2 s between the voltage steps. Patch-clamp recordings were analyzed and plotted with Igor Pro 5 software program (Wavemetrics, Portland, OR, USA).

### 2.7. Real-Time PCR

INS-1 cells were treated with 100 µM cyanidin for 24 h and RNA extracted for real-time PCR analysis of rat Insulin (Ins), glucose transporter type 2 (GLUT2), glucokinase (GK), ATP-sensitive K^+^ channel (Kir_6.2_), and l-type Ca^2+^ channel (Cav_1.2_) genes. Total RNA was extracted using RNAqueous^®^ Total RNA Isolation Kit (Ambion, Austin, TX, USA), based on the manufacturer’s instructions and purified with DNase 1 treatment. The cDNA was synthesized from 1 µg of total RNA using iScript^TM^ cDNA Synthesis Kit (Bio-Rad, Richmond, CA, USA). Real-time PCR was performed using SsoAdvanced^TM^ Universal SYBR^®^ Green Supermix (Bio-Rad, Richmond, CA, USA). Quantification was normalized to the β-actin gene. Experiments were repeated three times. 

The primers (forward/reverse [5′–3′]) were: CACCCAAGTCCCGTCGTGAAGT/GATCCACAATGCCACGCTTCTG (Ins); GAAATTCAAGAAGCGAAAAG/CCTGCTGTCACTCTGGTAGTAG (Cav_1.2_); TAAGGGGCACTGAGGACATC/TGCCAGCTGTCTGAAAAATG (GLUT2); AAGGGAACAACATCGTAGGA/CATTGGCGGTCTTCATAGTA (GK); TCCACCAGGTAGACATCCC/TAGGAGCCAGGTCGTAGAG (Kir_6.2_); GCGTGACATCAAAGAGAAG/ACTGTGTTGGCATAGAGG (β-actin).

### 2.8. Data Analysis

The results of insulin secretion, intracellular Ca^2+^ signal, and real-time PCR were expressed as mean ± standard error of the mean (S.E.M.) from the three independent experiments (*n* = 3). Patch-clamp recordings were expressed as representative current–voltage relationship (I/V) for VDCC channels from 24 to 31 cells from each group. The average current amplitude for cyanidin and control and the average traces for cyanidin and diazoxide were compared using unpaired Student’s *t*-test. Multiple group comparisons for cyanidin and control experiments including insulin secretion, intracellular Ca^2+^ signal and real-time PCR were carried out using one-way analysis of variance (ANOVA), followed by Tukey’s post hoc test (SPSS, Chicago, IL, USA). *p* < 0.05 was considered to be statistically significant.

## 3. Results

### 3.1. Intracellular Detection of Cyanidin

**Figure 1:**
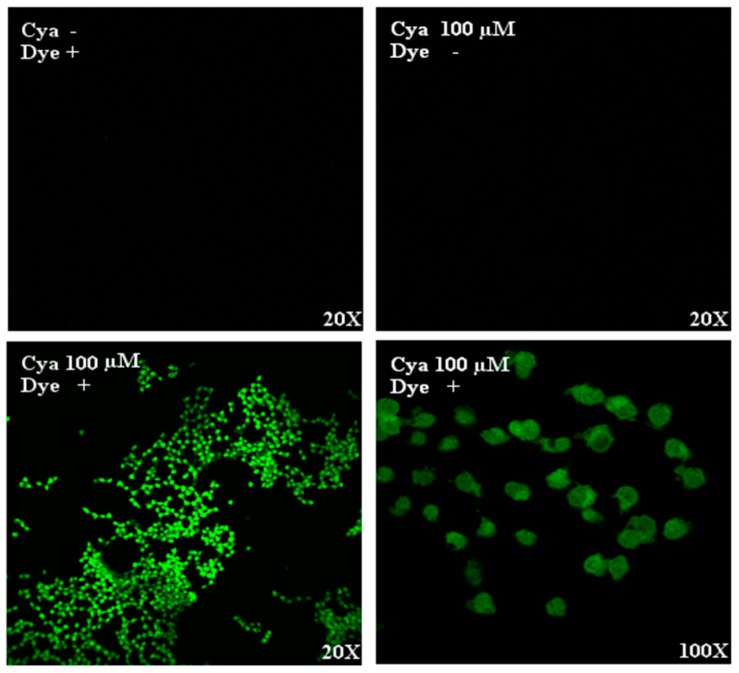
Localization of cyanidin in INS-1 cells. Confocal laser scanning microscopy after treatment of cells with 100 µM cyanidin for 10 min followed by Naturstoff reagent A showed the diffusion into the intracellular space.

To determine whether cyanidin could diffuse across the plasma membrane, Naturstoff reagent A was used. We detected the presence of cyanidin in the intracellular space 10 min after stimulation of cells and treatment with Naturstoff reagent A, but not after cyanidin or reagent alone (Figure 1). The presence of fluorescence in the cytoplasm indicates that cyanidin diffuses across the plasma membrane and promote its effect from the intracellular space.

### 3.2. Cyanidin Stimulates Insulin Secretion

**Figure 2:**
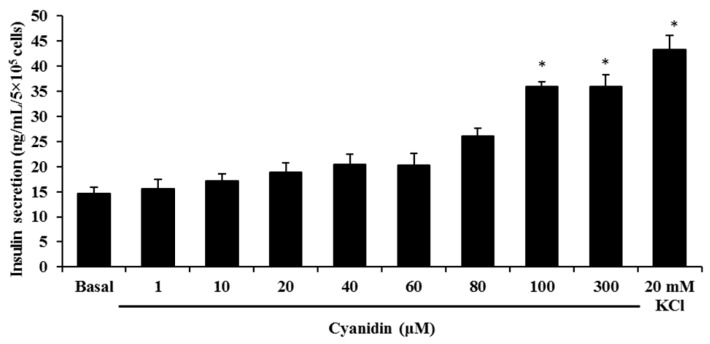
Treatment of INS-1 cells with 1–300 µM cyanidin increased insulin secretion. Results are expressed as mean ± S.E.M. from the three independent experiments; * *p* < 0.05 when compared with basal.

Next, we stimulated cells with increasing concentrations of cyanidin to examine whether it could stimulate insulin secretion. The results showed that cyanidin (1–300 µM) markedly increased insulin release from INS-1 cells (Figure 2). The calculated EC_50_ for cyanidin was 80.08 ± 2.11 µM. The maximum secretion was achieved with 100 µM cyanidin that caused a 2.5-fold increase over basal, whereas 20 mM KCl resulted in a 3-fold increase.

### 3.3. Cyanidin Increases Intracellular Ca^2+^ Concentration

**Figure 3:**
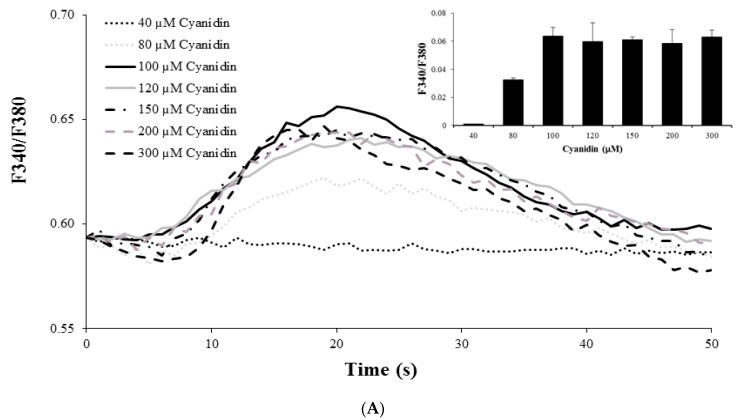

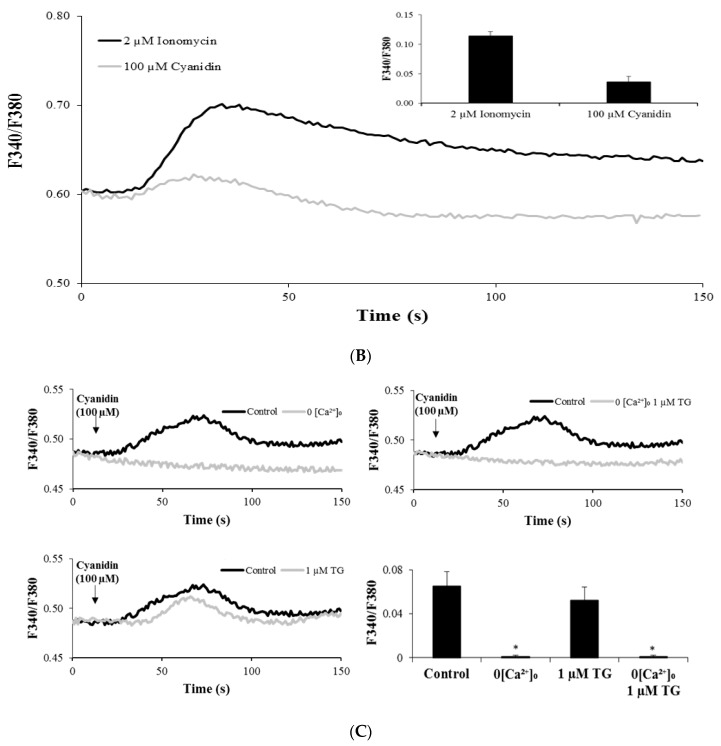
(**A**) Stimulation of cells with 40–300 µM cyanidin increased intracellular Ca^2+^ in a concentration-dependent manner; (**B**) 100 µM Cyanidin resulted in a 33–35% increase in intracellular Ca^2+^ compared to 2 µM ionomycin; (**C**) Removal of extracellular Ca^2+^ abolished the effect of cyanidin, but not after endoplasmic reticulum (ER) store depletion with thapsigargin. Under extracellular Ca^2+^ free and store depletion conditions, the cyanidin response is also abolished. Results are presented as average traces or mean + S.E.M.; *n* = 89–162 cells/group from the three independent experiments; * *p* < 0.05 when compared with the control.

Since elevations in intracellular Ca^2+^ are a requirement for insulin secretion, we examined whether cyanidin would induce Ca^2+^ signals. Stimulation of cells with cyanidin (80 to 300 µM) increased intracellular Ca^2+^ in a concentration-dependent manner (Figure 3A). The greatest increase was observed with 100 µM cyanidin. In comparison, cyanidin at 100 µM caused (33–35%) less Ca^2+^ increase than 2 μM ionomycin (Figure 3B). To investigate the Ca^2+^ sources for the cyanidin responses, experiments were performed under extracellular Ca^2+^ free conditions and/or after depletion of intracellular Ca^2+^ stores in the endoplasmic reticulum (ER). Removal of extracellular Ca^2+^ abolished the response to 100 µM cyanidin, but not after store depletion with thapsigarin, a Ca^2+^ ATPase pump inhibitor in the ER. Cyanidin also failed to increase intracellular Ca^2+^ under both Ca^2+^ free and store depletion conditions (Figure 3C).

### 3.4. Cyanidin Increases Intracellular Ca^2+^ via l-Type Voltage-Dependent Ca^2+^ Channels

**Figure 4:**
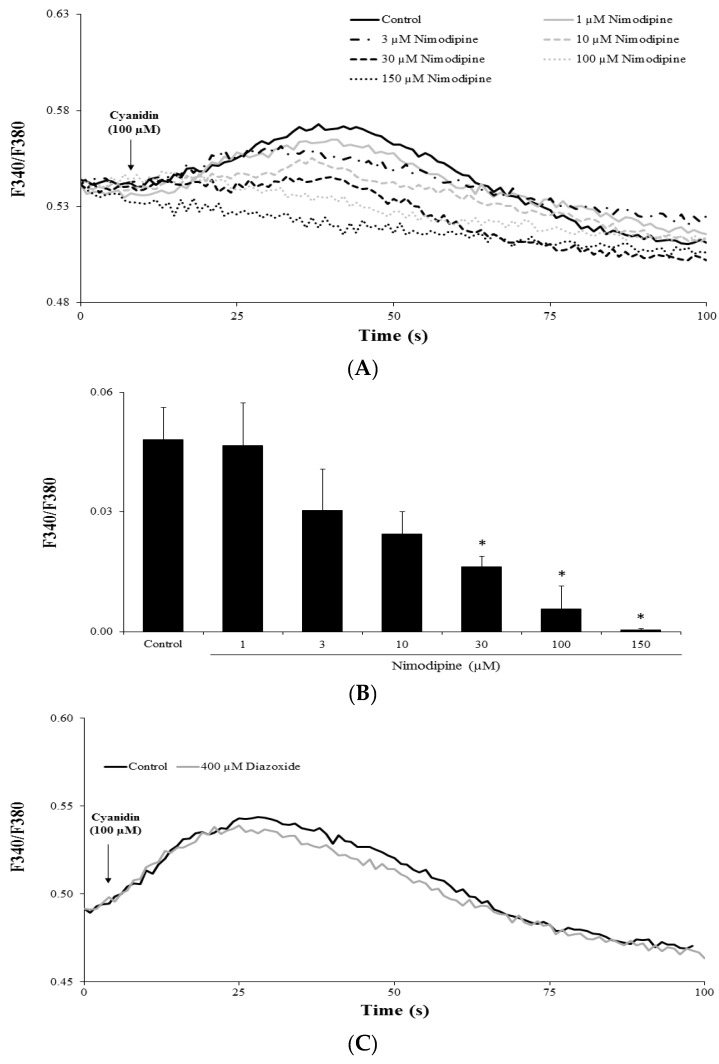
Voltage-dependent Ca^2+^ channels mediate the effect of cyanidin. (**A**,**B**) Pretreatment of cells with the l-type Ca^2+^ channel blocker nimodipine (1–100 µM) inhibited the intracellular Ca^2+^ signals by cyanidin in a concentration-dependent manner; (**C**) Pretreatment of cells with 400 µM diazoxide, a K_ATP_ activator, failed to inhibit the responses to cyanidin. Results are presented as average traces or mean + S.E.M.; *n* = 91–174 cells per group from the three independent experiments. * *p* < 0.05 when compared with the control.

In pancreatic β-cells, VDCCs are responsible for extracellular Ca^2+^ influx. Hence, we performed experiments with the l-type Ca^2+^ channel blocker nimodipine to test whether this was the case for cyanidin. Pretreatment of cells with 30–150 µM nimodipine inhibited the cyanidin-induced Ca^2+^ increases in a concentration-dependent manner (Figure 4A,B). To exclude the effect of cyanidin on K_ATP_ channels, cells were pretreated with diazoxide, which opens this particular channel. As shown in Figure 4C, diazoxide failed to inhibit the Ca^2+^ signals.

### 3.5. Cyanidin Directly Activates l-Type Voltage-Dependent Ca^2+^ Channels

**Figure 5:**
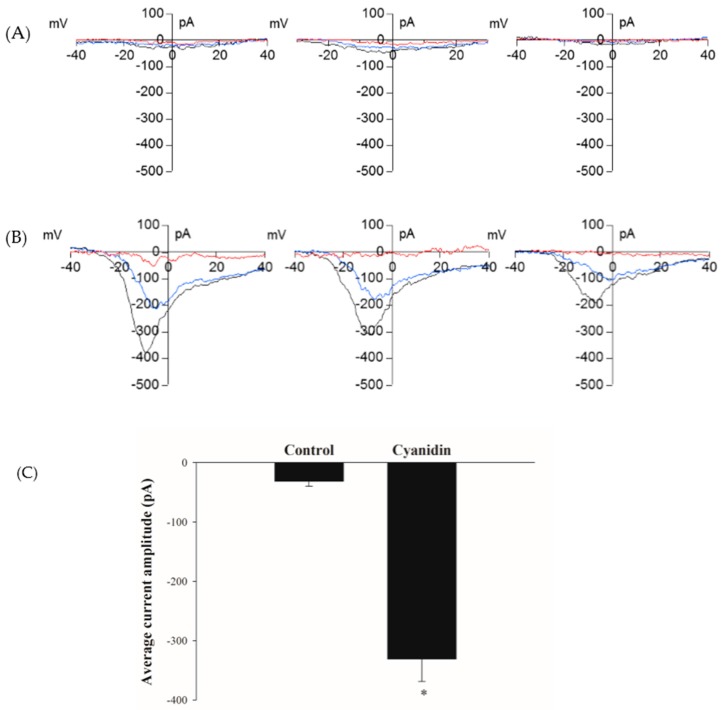
Cyanidin activates voltage-dependent Ca^2+^ channels. (**A**) Representative current–voltage relationships (I/V) from single cells perfused with control DMSO; (**B**) Representative current–voltage relationships (I/V) from single cells perfused with 100 µM cyanidin. The red (at break-in), blue (during), and black (fully developed) lines represent the current development; (**C**) Average inward peak current amplitude from control and 100 µM cyanidin groups. Results are represented as mean – S.E.M. (*n* = 24–31 cells/group). * *p* < 0.05 when compared with the control.

The Ca^2+^ imaging experiments revealed that l-type VDCCs were responsible for the increase in intracellular Ca^2+^. Therefore, we tested whether it could activate these channels directly using the patch-clamp technique. Perfusion of single cells with 100 µM cyanidin in the pipette solution resulted in the development of currents with the characteristics of VDCCs, but not in control cells perfused with 0.1% DMSO (Figure 5A,B). The peak current amplitude obtained from control and cyanidin-treated cells are shown in Figure 5C.

### 3.6. Up-Regulation of Insulin Secretion Genes by Cyanidin

**Figure 6:**
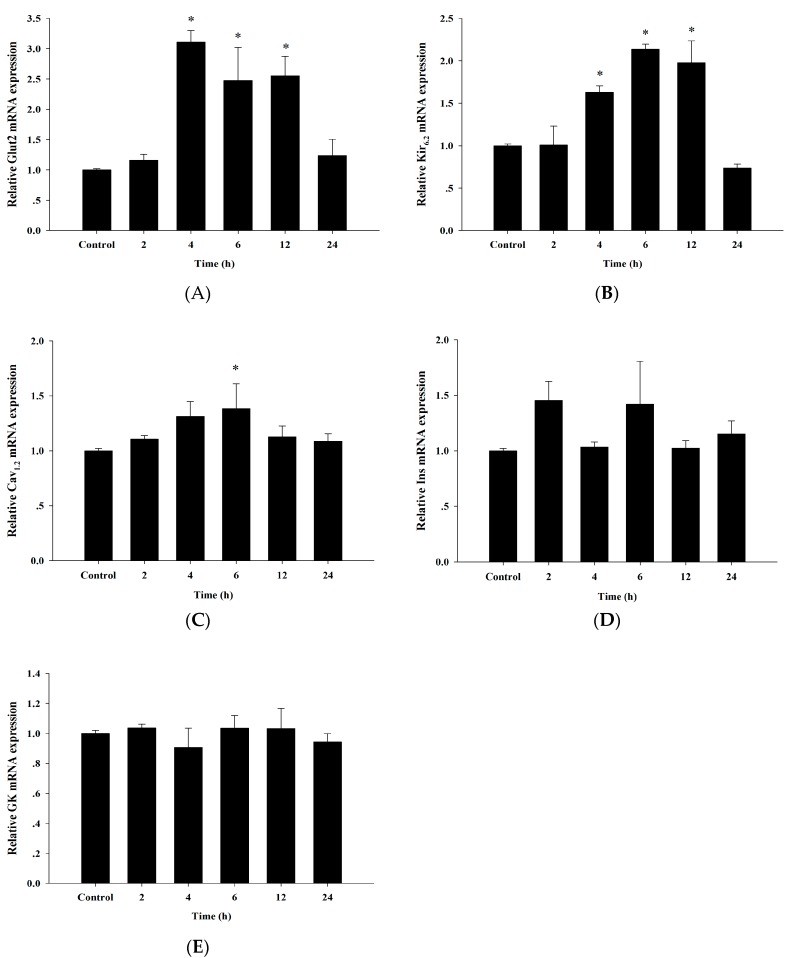
Effect of cyanidin on the mRNA levels of insulin secretion genes including Glut2; Glucose transporter 2 (**A**), Kir_6.2_; Potassium channel 6.2; (**B**), Cav_1.2_; Voltage-dependent Ca^2+^ channel 1.2; (**C**), Ins; Insulin; (**D**), and GK; Glucokinase; (**E**). All data normalized to β-actin expression. Results are expressed as mean ± S.E.M. from the three independent experiments; * *p* < 0.05 when compared with the control.

In addition to insulin secretion, we tested whether cyanidin regulated the expression of genes involved in the glucose-induced insulin secretion response. This would suggest a potential role in this mechanism. Treatment of cells with 100 µM cyanidin for 2, 4, 6, 12, and 24 h up-regulated the expression of the GLUT2, Kir_6.2_, and Cav_1.2_ genes (Figure 6). However, cyanidin did not impact the expression of the Insulin and glucokinase genes.

## 4. Discussion

The insulin secreting activity of cyanidin has been demonstrated in pancreatic β-cells [16]. Our findings revealed that its mechanism of action is linked to intracellular Ca^2+^ signaling via l-type voltage-dependent Ca^2+^ channels. They are also consistent with reports of other flavonoids such as quercetin and quercetin-3-rutinoside (rutin) that stimulate insulin secretion using Ca^2+^ signaling [19,20]. Increases in intracellular Ca^2+^ can be due to influx across the plasma membrane and/or release from the ER [21]. Our results clearly showed that the increase in intracellular Ca^2+^ was due to influx, as store depletion did not alter the cyanidin response. Furthermore, the l-type VDCC blocker nimodipine abolished the Ca^2+^ signals to indicate the pathway for Ca^2+^ influx. In support of this finding, the patch-clamp data confirmed the activation of channels. The opening of l-type Ca^2+^ channels represents the final common pathway for insulin secretion in pancreatic β-cells. l-type Ca^2+^ channels comprise five subunits, including α_1_, α_2_, β, γ, and δ subunits [22]. It has been recognized that Bay K 8644, a calcium channel agonist, specially binds to l-type Ca^2+^ channel α_1_ subunits with high affinity [22]. Our findings are similar to those reported for quercetin which stimulates insulin secretion through activation of l-type VDCCs [19,23]. However, Bardy et al. suggest that quercetin interacts with the l-type Ca^2+^ channel at a different site from that of Bay K 8644, an l-type Ca^2+^ channel agonist [19]. Additional studies are required to ascertain the binding sites of cyanidin on the l-type Ca^2+^ channel. Other phytochemical compounds such a *p*-methoxycinnamic acid [24] stimulate insulin secretion by increasing Ca^2+^ influx through VDCCs. This mechanism now appears to be universal among the flavonoids. In our study, we also tested whether cyanidin functions as sulfonylureas, which induces the closure of K_ATP_ channels leading to membrane depolarization, Ca^2+^ influx, and insulin secretion [25]. We found that diazoxide, a K_ATP_ channel opener, did not impact the effect of cyanidin on Ca^2+^ signals which is in agreement with genistein [26] and *p*-methoxycinnamic acid [24]. Other data have shown the effect of cyanidin on glucose-induced insulin secretion in pancreatic β-cells (INS-1 823/13). It found that cyanidin at a concentration of 50 μg/mL enhanced glucose-induced insulin release at 10 mM glucose [16]. It suggests that cyanidin may improve pancreatic β-cells which defect in the response to insulin secretion to glucose. 

In regard to its intracellular localization, flavonoids are absorbed into cells by passive diffusion and membrane transport [27]. We detected the presence of cyanidin in the cytosol of β-cells that is consistent with reports in HaCat human keratinocytes, human embryonic fibroblasts (NHF), uterine carcinoma (HeLa S3), and intestinal (CaCo-2) cells [20,28]. Quercetin enters Caco-2 cells and human embryonic kidney (HEK 293) cells through passive diffusion [29,30]. Faria et al. proposed that cyanidin-3-glucoside crosses the membrane of cerebral capillary endothelial (RBE-1) cells via glucose transporter-1 (GLUT1) [31]. It is hypothesized that hydrophilic flavonoid glucosides are transported across the membrane via glucose transporters, whereas passive diffusion of aglycone flavonoids occurs. It is possible that passive diffusion is involved in cellular uptake of cyanidin in pancreatic β-cells. Additional immunohistochemical stains together with Naturstoff reagent A might be required to identify the specific localization of cyanidin in the cells such as nucleus or plasma membrane l-type Ca^2+^ channel. Our gene expression analysis during cyanidin stimulation reports a down-regulation of genes associated with insulin secretion in diabetic animal models [32,33,34]. In this respect, chronic hyperglycemia leads to oxidative stress and gradual loss of pancreatic β-cell gene expression associated with impaired glucose-stimulated insulin secretion [35]. Administration of the glucose metabolite methylglyoxal reduces glucose uptake into β-cells due to down-regulation of the GLUT2 gene [36,37]. In regards to flavonoids, naringenin and quercetin increase GLUT2 gene expression to improve glucose-induced insulin secretion and glucose sensitivity [38]. In addition to GLUT2, the gene expression analysis during cyanidin stimulation demonstrated up-regulation of Kir_6.2_ and Cav_1.2_. The expression of Cav_1.2_ (a subunit of l-type VDCC) is crucial for insulin secretion because it promotes Ca^2+^ influx [39], whereas gene down-regulation reduces the glucose-induced secretion [40]. Our findings revealed that cyanidin activates VDCCs to promote Ca^2+^ influx, insulin secretion, and the expression of the Cav_1.2_ gene. The K_ATP_ channel comprises two subunits: the K^+^ inwardly rectifying (Kir_6.2_) and sulfonylurea receptor (SUR1). Hyperglycemia decreases Kir_6.2_ mRNA levels in rat pancreatic islets as well as in INS-1 cells [41] and reduces Ca^2+^ influx and insulin secretion from HIT-T15 cells [34]. Although cyanidin did not stimulate insulin secretion via the K_ATP_ dependent pathway, it might enhance glucose-induced insulin secretion by increasing Kir_6.2_ expression. Therefore, the up-regulation of genes controlled by cyanidin might be important to increase insulin sensitivity and insulin secretion. Further experiments are required to clarify the effect of cyanidin on these genes expression related to glucose-stimulated insulin secretion (GSIS) under dysfunction of pancreatic β-cells. Finally, there is accumulating evidence that cyanidin can stimulate insulin secretion [16], and inhibit protein glycation [15], intestinal α-glucosidase, and pancreatic α-amylase [11]. Our studies are consistent with other studies demonstrating that the concentration range of cyanidin (0.5–100 µM) exerted their preventive effects without noticeable toxicity in the cell line models [42,43,44]. However, plasma concentration of cyanidin and its derivatives can be detected in the low micromolar range [45]. At present, the aspects of bioavailability and the cellular concentration of cyanidin, especially in pancreatic β-cells, hepatocytes, and adipose tissue, are still not fully documented. To overcome bioavailability issues of cyanidin, encapsulation techniques might be a useful platform for the possible translation to clinical evaluation.

## 5. Conclusions

**Figure 7:**
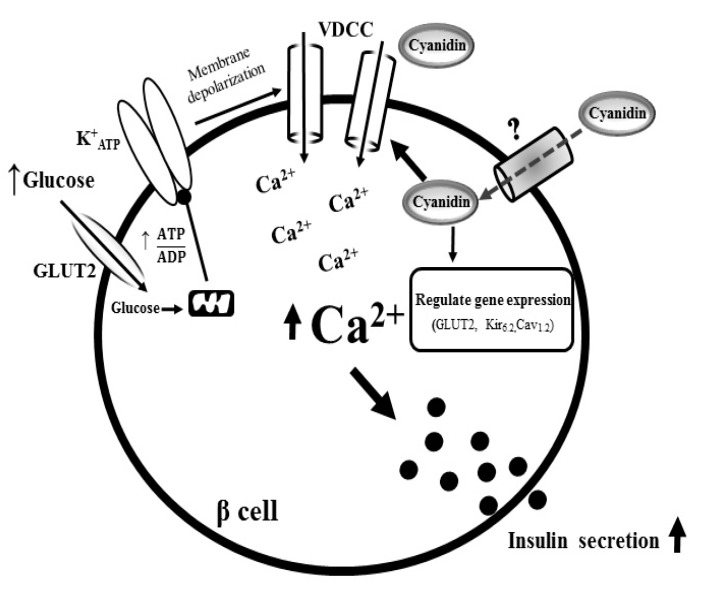
Proposed diagram for the mechanism of action of cyanidin. In pancreatic β-cells, cyanidin diffuses across the plasma membrane, leading to the activation of l-type VDCCs. Consequently, it promotes Ca^2+^ influx which stimulates insulin secretion. The increases in intracellular Ca^2+^ also up-regulate the expression of the GLUT2, Kir_6.2_ and Cav_1.2_ genes.

The results from our study revealed that cyanidin diffuses across the plasma membrane, leading to the activation of l-type VDCCs. This effect promotes Ca^2+^ influx which stimulates insulin secretion from pancreatic β-cells (Figure 7). Cyanidin also up-regulated the expression of the GLUT2, Kir_6.2_ and Cav_1.2_ genes that could have potential implications on glucose-induced insulin secretion, glucose homeostasis, and diabetes.

## Abbreviations

GLUT1	Glucose transporter 1
GLUT2	Glucose transporter 2
SUR1	Sulfonylurea receptor
K_ATP_	ATP-sensitive K^+^ channels
VDCCs	Voltage-dependent Ca^2+^ channels
MG	Methylglyoxal
Fura-2AM	Fura-2 acetoxymethyl ester
KRB	Krebs-Ringer bicarbonate buffer
RIA	Radioimmunoassay
Vh	holding potential
GK	Glucokinase
Cav_1.2_	l-type Ca^2+^ channel
Ins	Insulin
I/V	Current–voltage relationship
ER	Endoplasmic reticulum
Rutin	Quercetin-3-rutinoside
